# Exploratory analysis of one versus two-day intermittent fasting protocols on the gut microbiome and plasma metabolome in adults with overweight/obesity

**DOI:** 10.3389/fnut.2022.1036080

**Published:** 2022-10-26

**Authors:** Alex E. Mohr, Paniz Jasbi, Devin A. Bowes, Blake Dirks, Corrie M. Whisner, Karen M. Arciero, Michelle Poe, Haiwei Gu, Eric Gumpricht, Karen L. Sweazea, Paul J. Arciero

**Affiliations:** ^1^College of Health Solutions, Arizona State University, Phoenix, AZ, United States; ^2^School of Molecular Sciences, Arizona State University, Tempe, AZ, United States; ^3^Center for Health Through Microbiomes, The Biodesign Institute, Arizona State University, Tempe, AZ, United States; ^4^Human Nutrition and Metabolism Laboratory, Department of Health and Human Physiological Sciences, Skidmore College, Saratoga Springs, NY, United States; ^5^Center for Translational Science, Florida International University, Port St. Lucie, FL, United States; ^6^Isagenix International, LLC, Gilbert, AZ, United States; ^7^School of Life Sciences, Arizona State University, Tempe, AZ, United States

**Keywords:** gut microbiome, intermittent fasting, metabolome, caloric restriction, obesity, weight loss, gastrointestinal symptoms, protein pacing

## Abstract

Nutritional interventions are a promising therapeutic option for addressing obesity and cardiometabolic dysfunction. One such option, intermittent fasting (IF), has emerged as a viable alternative to daily caloric restriction and may beneficially modulate body weight regulation and alter the gut microbiome (GM) and plasma metabolome. This secondary analysis of a larger, registered trial (ClinicalTrials.gov ID: NCT04327141) examined the effect of a four-week intervention comparing one vs. two-consecutive days of IF in combination with protein pacing (IF-P; 4-5 meals/day, >30% protein/day) on the GM, the plasma metabolome, and associated clinical outcomes in overweight and obese adults. Participants (*n* = 20) were randomly assigned to either a diet consisting of one fasting day (total of 36 h) and six low-calorie P days per week (IF1-P, *n* = 10) or two fasting days (60 h total) and five low-calorie P days per week (IF2-P, *n* = 10). The fecal microbiome, clinical outcomes, and plasma metabolome were analyzed at baseline (week 0) and after four weeks. There were no significant time or interaction effects for alpha diversity; however, baseline alpha diversity was negatively correlated with percent body fat change after the four-week intervention (*p* = 0.030). In addition, beta-diversity for both IF groups was altered significantly by time (*p* = 0.001), with no significant differences between groups. The IF1-P group had a significant increase in abundance of Ruminococcaceae *Incertae Sedis* and *Eubacterium fissicatena group* (*q* ≤ 0.007), while the IF2-P group had a significant increase in abundance of Ruminococcaceae *Incertae Sedis* and a decrease in *Eubacterium ventriosum group* (*q* ≤ 0.005). The plasma metabolite profile of IF2-P participants displayed significant increases in serine, trimethylamine oxide (TMAO), levulinic acid, 3-aminobutyric acid, citrate, isocitrate, and glucuronic acid (*q* ≤ 0.049) compared to IF1-P. Fecal short-chain fatty acid concentrations did not differ significantly by time or between groups (*p* ≥ 0.126). Interestingly, gastrointestinal symptoms were significantly reduced for the IF2-P group but not for the IF1-P group. Our results demonstrate that short-term IF modestly influenced the GM community structure and the plasma metabolome, suggesting these protocols could be viable for certain nutritional intervention strategies.

## Introduction

Obesity continues to be highly prevalent in the United States. In 40% of the adult population, increased adiposity is tightly intertwined with cardiometabolic perturbation and is a primary comorbidity of major chronic disease (1). Non-invasive, nutrition-based approaches are the most widely utilized, feasible, and effective options to reduce body fat and support healthy lifestyle changes (2). To promote energy deficit, a daily caloric restriction of 10–40% is a common intervention in weight loss research and clinical practice (2, 3). However, such a dietary prescription may not be sustainable for most individuals in relation to long term adherence due to behavioral, psychosocial, and environmental factors (4). As a dietary regimen growing in scientific, clinical, and public interest, intermittent fasting (IF) is an alternative approach that holds promise for promoting healthy body weight and metabolic functioning (5). Intermittent fasting encompasses eating patterns in which individuals go extended periods (e.g., 16–60 h) with little or no energy intake and intervening periods of normal food intake (6). A common and well-tolerated IF regimen that has been implemented in long-term weight loss and weight maintenance interventions is modified fasting regimens, which allows the consumption of 20–25% of energy needs on scheduled fasting days. For example, normal feeding for five to six days and fasting for one to two days weekly (5).

In comparison to complete fasting, nutritionally supported fasts have been reported to improve glycemic control and reduce hunger ratings (7). Such nutritional regimens have offered a new frontier for research and pose a potential dietary framework for treating obesity and metabolic disease. Indeed, encouraging results have been reported for improved body composition, energy expenditure, and cardiometabolic markers (e.g., fasting plasma lipids, insulin, and glucose) (8–10). The lack of attention to the non-fasting days/period in previous investigations is a significant oversight and may impact the effectiveness of the IF. Previous research has consistently shown a higher protein diet (>30%), evenly distributed throughout the day (4–5 meals/day), moderate-carbohydrate (<40%) and low-glycemic index (<50), known as protein pacing (P) during feeding days, with and without caloric restriction, significantly enhances body composition, energy expenditure, and cardiometabolic health (8–10) and should be considered in the overall weight loss regimen.

Another emerging research area is the influence of nutrition on the composition and function of the microbes harbored in the gastrointestinal (GI) tract, known as the gut microbiome (GM). This research suggests significant shifts in microbial composition, function, and metabolic output in response to dietary changes (11, 12). Accordingly, GM dysfunction has been linked to obesity (13, 14). Diets that drive increased adiposity are generally high in energy and fat while low in fiber and diet quality. Obesogenic diets may promote luminal mucus degradation and pathogen encroachment (15), decrease community diversity and beneficial taxa (16), and malign GM function and metabolic output (17, 18). As a dense microbial bioreactor, gut microorganisms have tremendous functional capacity, producing an array of metabolites that have varying effects on host health (19). Analogously, the metabolome is defined as the complete suite of small molecules present in a biological system and is also modified by similar host-associated characteristics, including the GM (20). Therefore, any potential interactions between the GM and metabolome may be of significant interest in obesity and obesity-related conditions.

Recent, well-performed clinical trials implementing daily caloric restriction have focused on the effects of weight loss on the GM (21–23). However, evidence also demonstrates that IF significantly impacts the GM composition and function (24). Much clinical research has employed time-restricted eating regimens in various populations, revealing significant dissimilarity compared to control but no differences in taxa abundance (25–28). More recent work utilizing IF has shown shifts in GM community metrics and gut-related metabolites compared to control in patients diagnosed with metabolic syndrome (29, 30). However, the control group participants were asked to maintain a routine diet without specific dietary instructions. Intermittent fasting research could greatly benefit by better establishing well-controlled comparison groups and carefully considering nutritional quality during feeding periods. Moreover, the evaluation of different fasting durations is sparse in the literature.

To the best of our knowledge, the direct comparison of different IF durations emphasizing dietary control and quality of nutrition consumed during both fasting and feeding days in a randomized study design assessing the GM has not yet been conducted. Therefore, as part of a larger clinical weight-loss trial, this exploratory analysis compared the effect of a four-week intervention of one-day (IF1-P) versus two-day (IF2-P) fasting with protein pacing on the GM and the plasma metabolome of overweight and obese adults. As a secondary aim, we examined self-reported GI symptomology between groups. We hypothesized that there would be a significant shift in GM community metrics and the plasma metabolome between IF1-P and IF2-P.

## Materials and methods

### Participant characteristics and study design

**FIGURE 1:**
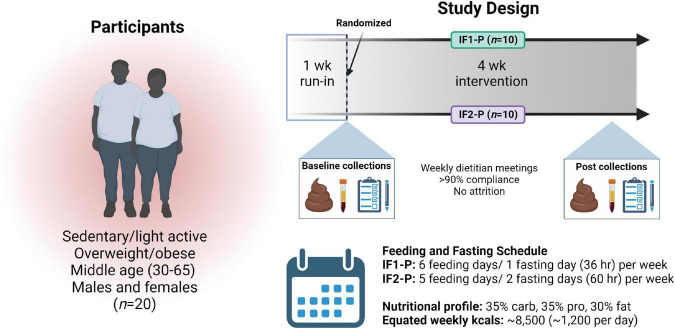
Overview of study design. After enrollment, a one-week run-in period, and randomization, 20 overweight and obese males and females followed an intermittent fasting, protein pacing-based weight loss diet consisting of one (IF1-P, *n* = 10) or two (IF2-P, *n* = 10) fasting days per week for four weeks equated for weekly energy and macronutrient intake. Clinical data and fecal and blood samples were collected at baseline and after week four. “Created with BioRender.com”.

Participant samples and data used in the present analysis were procured from a larger, registered clinical trial (ClinicalTrials.gov ID: NCT04327141). Study design, clinical outcomes, and participant characteristics have been reported previously (31). Briefly, participants were healthy, non-smoking, sedentary/lightly active, males and females with overweight/obesity. Participants taking antibiotics, antifungals, or probiotics within the previous two months were excluded. This study was approved by the Institutional Review Boards of Skidmore College, NY, USA, and Arizona State University, AZ, USA, and all participants provided written informed consent before study enrollment. A total of 20 participants were enrolled and completed a one-week run-in period maintaining stable body weight and physical activity level. Following this period, participants were then randomly assigned to a modified fasting regimen consisting of one fasting day (total of 36 h) and six P feeding days per week (IF1-P, *n* = 10) or two fasting days (60 h total) and five P feeding days per week (IF2-P, *n* = 10) (Figure 1). This dietary regimen has been previously shown to be effective for weight loss and has high compliance rates (9, 10, 31). Detailed guidelines were provided to participants at weekly meetings with a registered dietitian nutritionist (RDN). On the fasting days, participants were supplied nutritional support providing approximately 400-500 kcals per day and comprised of various supplements and snacks, as previously described (31). On P feeding days in the IF1-P group, females consumed four meals daily, providing 1,350 kcals, and males consumed five meals daily, providing 1,700 kcals [for a full description of both fasting and feeding days, see (31)]. The nutritional profile was 35% carbohydrate, 35% protein, and 30% fat, consisting of two liquid meal replacements (Isagenix International, LLC, Gilbert, AZ, USA), one whole food dinner, and one or two snacks (female and male, respectively). To ensure an equivocal macronutrient profile and weekly energy intake as IF1-P (∼8,500 kcals), IF2-P followed a similar dietary regimen consisting of four meals a day for females providing 1,500 kcals and five meals a day for males providing 1,850 kcals. The same nutritional profile of 35% protein, 35% carbohydrate, and 30% fat was used and consisted of two liquid meal replacements, one whole food dinner, and one or two snacks (female and male, respectively). Throughout the study, the RDN and investigators ensured adherence to the IF-P regimens via weekly meetings, detailed written instructions, and daily communication (e.g., email, text, and mobile phone). Moreover, two-day food diary analyses were conducted, as well as weekly inspection of dietary intake, distribution of weekly meal/supplement containers, and return of empty packets and containers.

### Gastrointestinal symptom rating scale

Participants completed the 15-question gastrointestinal symptom rating scale (GSRS) (32) at baseline and the end of the four-week intervention. Briefly, each question is rated on a 7-point Likert scale (1 = absent; 2 = minor; 3 = mild; 4 = moderate; 5 = moderately severe; 6 = severe and 7 = very severe) and recalled from the previous week. Questions include symptoms related to upper abdominal pain, heartburn, regurgitation (acid reflux), empty feeling in the stomach, nausea, abdominal rumbling, bloating, belching, flatulence, and questions on defecation. The GSRS questionnaire provides explanations of each symptom, is understandable, and has reproducibility for measuring the presence of GI symptoms (33). In our analysis, a score of ≥2 (minor) was defined as symptom presence, and a score ≥4 (moderate) was defined as moderate symptom presence. Furthermore, to better categorize symptom location, bloating, flatulence, constipation, diarrhea, and defecation urgency were classified as lower GI symptoms, and nausea, heartburn, regurgitation, upper abdominal pain, empty feeling in the stomach, and belching were classified as upper GI symptoms. Total scores were also generated for both overall symptom and moderate symptom presence.

### Fecal sample collection and processing

Participants were instructed to provide stool samples at baseline and after week four of the intervention. The entire bowel movement was collected and transported within 24 h of defecation to the Skidmore College laboratory using a cooler and ice packs, and frozen at −80^°^C. Samples were then sent to ASU (Phoenix, AZ, USA) overnight on dry ice for analysis, where they were thawed at 4^°^C and processed. Wet weight was recorded to the nearest 0.01 g after subtracting the weight of fecal collection materials. Stool samples were then rated according to the Bristol Stool Scale (BSS) (34), homogenized in a stomacher bag, and the pH was measured (Symphony SB70P, VWR International, LLC., Radnor, PA, USA). DNA extraction was performed using the DNeasy PowerSoil Pro Kit (Cat. No. 47016, Qiagen, Germanton, MD, USA), per the manufacturer’s instructions. DNA concentration and quality were quantified using the NanoDrop™ OneC Microvolume UV-Vis Spectrophotometer (Thermo Scientific™, Waltham, MA, USA) according to manufacturer instructions. The OD_260_/OD_280_ ratio of all samples was ≥1.80 (demonstrating DNA purity).

### Fecal short-chain fatty acid analysis

High-performance liquid chromatography (HPLC; Shimadzu, Kyoto, Japan) was used to quantify short-chain fatty acid (SCFA) concentrations in fecal samples, as reported previously with slight modifications (35). Briefly, for each sample 300 mg fecal matter was added to 6 mL of 18Ω deionized water in a 15 mL falcon tube and vortexed at 3,200 rpm for 10 min at 24^°^C. This mixture was then centrifuged at 4,000 rpm for 10 min at 24^°^C and then filtered/sterilized through a 0.22 μm syringe filter. One mL of the resulting supernatant was then used as the analytical sample for the HPLC analysis. The organic acids were separated and identified using a Bio-Rad column (Aminex HPX-87H) with an Agilent diode array detector at 65^°^C, 5 mM H_2_SO_4_ as mobile phase, 210 nm measurement wavelength, and a 0.6 mL/min flow rate that was increased to 0.8 mL/min at 60 min.

### Fecal microbiome analysis

The GM was assessed from the DNA extracted from the fecal collections at the Biodesign Institute at ASU (Tempe, AZ, USA). Amplification of the 16S rRNA gene sequence was completed in triplicate polymerase chain reactions (PCRs) using 96-well plates for GM composition. Barcoded universal forward 515F primers and 806R reverse primers containing Illumina adapter sequences, which target the highly conserved V4 region, were used to amplify microbial DNA (36, 37). These primers were selected as recommended by the Earth Microbiome Project (36, 37) and the National Institutes of Health Human Microbiome Project (38) to enhance reproducibility and comparability to other studies while obtaining broad coverage of Bacteria. PCR, amplicon cleaning, and quantification were performed as previously outlined (37). Equimolar ratios of amplicons from individual samples were pooled together before sequencing on the Illumina platform (Illumina MiSeq instrument, Illumina, Inc., San Diego, CA). Raw Illumina microbial data were cleaned by removing short and long sequences, sequences with primer mismatches, uncorrectable barcodes, and ambiguous bases using the Quantitative Insights into Microbial Ecology 2 (QIIME2) software, version 2021.8 (39).

16S rRNA gene sequencing produced 2,610,204 reads with a median of 50,609 per sample (range: 9,512 – 470,848). Paired-end, demultiplexed data were imported and analyzed using QIIME2 software. Upon examination of sequence quality plots, base pairs were trimmed at position 20 and truncated at position 240 and were run through DADA2 to remove low-quality regions and construct a feature table using amplicon sequence variants (ASVs.). All singleton reads were also removed from the dataset. Next, the ASV feature table was passed through the feature-classifier plugin (40), which was implemented using a naive Bayes machine-learning classifier, pre-trained to discern taxonomy mapped to the latest version of the rRNA database SILVA (138.1; 99% ASVs. from 515F/806R region of sequences) (41). A phylogenic tree was then constructed using the fragment-insertion plugin with the SILVA database. Based on the assessment of alpha rarefaction, a threshold of 6,407 sequences/sample was established and used to normalize samples for uneven sequencing depth for the subsequent diversity analyses (42). Alpha diversity was measured using Shannon (abundance and evenness of taxa present) and Faith’s phylogenetic difference (PD) (incorporates phylogenetic difference between taxa) indexes. Beta diversity was measured using the Bray-Curtis dissimilarity index. Predicted functional potential of the overall bacterial community was surveyed via the Phylogenetic Investigation of Communities by Reconstruction of Unobserved States 2 (PICRUSt2) algorithm (v2.4.2) (43). Pathway abundances were inferred based on structured pathway mappings of Enzyme Commission gene families to the MetaCyc database (44).

To provide an estimate of total bacterial biomass per sample (16S rRNA gene copies per gram of wet stool), DNA extracted from the fecal collections was assessed via quantitative polymerase chain reaction (qPCR) based on previously published methods (45, 46). Briefly, all 20 μL qPCR reactions contained 10 uL of 2X SYBR *Premix Ex Taq™* (Tli Rnase H Plus) (Takara Bio USA, Inc., San Jose, CA, USA), 0.3 μM (0.6 μL) of each primer (926F: AAACTCAAAKGAATTGACGG; 1062R: CTCACRRCACGAGCTGAC), 2 μL DNA template (or PCR-grade water as negative control), and 6.8 μL nuclease-free water (Thermo Fisher Scientific, Waltham, MA, USA). PCR thermal cycling conditions were as follows: 95^°^C for 5 min, followed by 35 cycles of 95^°^C for 15 s, 61.5^°^C for 15 s, and 72^°^C for 20 s, then hold at 72^°^C for 5 min, along with a melt curve of 95^°^C for 15 s, 60^°^C for 1 min, then 95*^o^*C for 1 s. Quantification was performed using a QuantStudio3 Real-Time PCR System by Applied Biosystems with QuantStudio Design and Analysis Software 1.2 from Thermo Fisher Scientific (Waltham, MA, USA). All samples were analyzed in technical replicates. For quality assurance and quality control, molecular negative template controls (NTC) consisting of PCR-grade water (Invitrogen, Waltham, MA, USA) along with positive controls created by linearized plasmids were run on every qPCR plate. Standard curves were run in triplicate and used for sample quantification, ranging from 10^7^ to 10^1^ copies/μL with a cycle threshold (CT) detection limit cutoff of 33. Reaction efficiency was approximately 101%, with a slope of -3.29 and *R*^2^ ≥ 0.99.

### Targeted plasma metabolomic analysis

For the plasma metabolomic analysis, a 12-h fasted venous blood sample (∼20 mL) was collected into EDTA-coated vacutainer tubes and centrifuged (Hettich Rotina 46R5) for 15 min at 2,500 RPM at 4^°^C. After separation, 2 mL of plasma was aliquoted and stored at −80^°^C. Samples were then sent to the Arizona Metabolomics Laboratory at ASU (Phoenix, AZ, USA) overnight on dry ice for analysis, where they were thawed at 4^°^C and processed. Briefly, 50 μL of plasma from each sample was processed to precipitate proteins and extract metabolites by adding 500 μL MeOH and 50 μL internal standard solution (containing 1,810.5 μM ^13^C_3_-lactate and 142 μM ^13^C_5_-glutamic acid). The mixture was vortexed (10 s) and stored for 30 min at –20^°^C, then centrifuged at 14,000 RPM for 10 min at 4^°^C. Supernatants (450 μL) were extracted and transferred to new Eppendorf vials and dried (CentriVap Concentrator; Labconco, Fort Scott, KS, USA). Samples were then reconstituted in 150μL of 40% phosphate-buffered saline (PBS)/60% acetonitrile (ACN) and centrifuged again at 14,000 RPM at 4^°^C for 10 min. Supernatants (100 μL) were transferred to an LC autosampler vial for subsequent analysis. Internal quality-control (QC) was performed by creating a pooled sample from all plasma samples and injected once every ten experimental samples to monitor system performance.

The highly reproducible targeted LC-MS/MS method used in the current investigation was modeled after previous studies (47–49). The specific metabolites included in our targeted detection panel are representative of more than 35 biological pathways most essential to central carbon metabolism, and have been successfully leveraged for the sensitive and broad detection of effects related to diet (50), disease (51), drug treatment (52), environmental contamination (53), and lifestyle factors (54). Briefly, LC-MS/MS experiments were performed on an Agilent 1290 UPLC-6490 QQQ-MS system (Santa Clara, CA, USA). Each sample was injected twice for analysis, 10 μL using negative ionization mode and 4 μL using positive ionization mode. Chromatographic separations were performed in hydrophilic interaction chromatography (HILIC) mode on a Waters Xbridge BEH Amide column (150 × 2.1 mm, 2.5 μm particle size, Waters Corporation, Milford, MA, USA). The flow rate was 0.3 mL/min, the auto-sampler temperature was maintained at 4^°^C, and the column compartment was set at 40^°^C. The mobile phase system was composed of Solvents A (10 mM ammonium acetate, 10 mM ammonium hydroxide in 95% H_2_O/5% ACN) and B (10 mM ammonium acetate, 10 mM ammonium hydroxide in 95% ACN/5% H2O). After the initial 1 min isocratic elution of 90% Solvent B, the percentage of Solvent B decreased to 40% at *t* = 11 min. The composition of Solvent B was maintained at 40% for 4 min (*t* = 15 min), and then the percentage of Solvent B gradually went back to 90% to prepare for the next injection.

The mass spectrometer was equipped with an electrospray ionization (ESI) source. Targeted data acquisition was performed in multiple-reaction-monitoring (MRM) mode. The LC-MS system was controlled by Agilent MassHunter Workstation software (Santa Clara, CA, USA), and extracted MRM peaks were integrated using Agilent MassHunter Quantitative Data Analysis software (Santa Clara, CA, USA).

### Statistical analysis

Gastrointestinal (GI) symptom scores were on the low end of the GSRS scale and not normally distributed; therefore, non-parametric statistical tests were applied. Individual scores between groups were assessed using a Mann-Whitney U test. Symptom prevalence (number of scores ≥2) and moderate symptom prevalence (≥4) for total, upper, and lower GI GSRS clusters were analyzed using contingency tables. Specifically, differences between IF1-P and IF2-P GI symptoms at baseline were compared using a Fisher’s Exact test, whereas pre-post values were compared with McNemar’s test. Stool weight, BSS, fecal pH, and SCFAs were assessed for normality with Q-Q plots and Shapiro-Wilk tests and log-transformed where appropriate. These were then tested for time and interaction (group × time) effects using linear-mixed effect (LME) models, with each participant included as a random effect. All tests were performed with a significance level of *p* < 0.05. Statistical analyses were performed using SPSS 27.0 (SPSS Inc., Chicago, IL, USA).

For analysis and visualization of the microbiome data, artifacts generated in QIIME2 were imported into the R environment (v4.1.2) using the *phyloseq* package (v1.38.0) (55). Before conducting downstream analyses, sequences were filtered to remove all non-bacterial sequences, including archaea, mitochondria, and chloroplasts. For beta diversity, a permutational analysis of variance (PERMANOVA) was conducted on Bray-Curtis dissimilarities using the Adonis test in the *vegan* package (v2.6.2) with 999 permutations. The PERMANOVA model incorporated the factors of time, individual, and interaction (group × time). A permutation test for homogeneity in multivariate dispersion (PERMDISP) was conducted using the “betadisper” function in the *vegan* package to compare dispersion. To support the Adonis analysis, the first principal coordinate of a principal coordinate analysis on the Bray-Curtis dissimilarity matrix was calculated and rank-transformed. A LME model was constructed on these values, using the *nLME* package (v3.1.153), testing the interaction effect of group and time with individual as a random effect. Beta diversity first-distances were also compared between groups, as previously described (56), by calculating the within-subject distance for paired samples (baseline vs. week 4) and testing for group distances (Wilcoxon rank-sum test). After assessing normality (Shapiro-Wilk’s tests), LME models were used to test the effect of time and the interaction of group and time with each participant included as a random effect on the alpha diversity metrics using the *nLME* package. Associations between baseline GM and adiposity were assessed with multiple regression models to explore potential differential diet responses using GM as a determinant. These associations were calculated using baseline alpha diversity metrics and change in percent body fat (post – pre values), accounting for age, sex, and baseline body mass index (BMI) covariates.

Estimates of bacterial biomass (qPCR data) were assessed for normality and entered into a LME model, as described above. With taxonomy, a ratio was calculated for two of the most predominant phyla, Firmicutes/Bacteroidota (FB ratio), log-transformed, and assessed via LME analysis. For differential abundance testing, analysis of compositions of microbiomes with bias correction (ANCOM-BC) was employed on GM taxa and PICRUSt2 output using the R package *ANCOMBC* (v1.4.0) (57). First, raw counts from the ASV table were filtered for any sequence not present in at least 30% of all samples, and a detection limit for the ANCOM-BC models was established at a value of 0.7 (tested at each phylogenic level). The same approach was followed on the predicted functional pathways from the PICRUST2 data. Based on the sample size, parallel-group design, and zero-inflation common to microbiome data, ANCOM-BC was implemented for each group separately, assessing the effect of time. Changes were calculated as the log2 fold change (log2FC) of abundance at week 4 versus baseline. Differentially abundance genera were also assessed for potential associations with adiposity by running Spearman rank correlation tests between change in centered log-ratio transformed taxa (post – pre abundance) and change in percent body fat (post – pre values). Where appropriate, false discovery rate (FDR) corrections were used to adjust for multiple hypothesis testing with a significance level of *q* < 0.05.

Univariate and multivariate analyses of plasma metabolites and metabolic ontology analysis were performed, and results were visualized using the MetaboAnalystR 5.0 (58). Human metabolomic data were mapped to the Kyoto Encyclopedia of Genes and Genomes (KEGG) human pathway library to analyze predicted states (59). The data were log_10_-transformed, and Pareto scaled to approximate normality prior to all analyses. A general linear model (GLM) was constructed with age, sex, time, and baseline BMI covariates to determine significantly affected metabolites by group intervention. Levene’s test was performed to detect significant homogeneity. Spearman rank correlation tests were performed using change in percent body fat and change in metabolites (post – pre values). Corrections were performed as indicated. An FDR correction was adjusted for multiple hypothesis testing with a significance level of *q* < 0.05.

## Results

### Participant characteristics

Baseline characteristics of the study participants of the IF1-P and IF2-P groups are displayed in Table 1. Age, sex, BMI, and physical activity level did not differ by group. The overall mean percentage of kilocalories from carbohydrate, protein and fat at baseline was 43.6 ± 12.8%, 16.5 ± 4.9%, and 39.9 ± 10.4%, respectively. Both carbohydrate and fat consumption were outside the acceptable macronutrient distribution range (AMDR) of 45–65% and 20–35%, respectively, whereas mean protein consumption was on the lower end of the AMDR range of 10–35% (60). The mean self-reported daily intake of sugar consumed was 103.3 ± 49.4 g/d. Mean daily consumption of dietary fiber for males (*n* = 6) and females (*n* = 14) was 25.9 ± 10.9 g/d and 17.0 ± 5.4 g/d, respectively, for which both fell below the AMDR for males (38 g/d) and females (25–26 g/d) (60). As previously described, both IF1-P and IF2-P similarly and significantly altered participants’ dietary energy and macronutrient intake (31). Briefly, total energy intake decreased by ∼40% (∼1,000 kcals/d) with no significant difference between groups. This reduction was due to significant decreases in dietary fat (-50 to 60 g/d) and carbohydrate (-138 to 152 g/d) intake. Protein intake increased significantly (21–25 g/d) in both groups. These macronutrient changes resulted in a distribution of 32–37% carbohydrate, 34–35% protein, and 28–34% fat. Moreover, dietary fiber intake significantly increased (8–12 g/d), whereas sugar (-57 to 77 g/d) and sodium (-1,500 to 2,000 mg/d) significantly decreased. As previously reported, both groups maintained similar physical activity and energy expenditure throughout the weight loss period (31).

**TABLE 1:** Baseline characteristics of study participants.

Variable	IF1-P (*n* = 10)	IF2-P (*n* = 10)	Total (*n* = 20)
Age	47.3 ± 10.0	52.0 ± 8.6	49.7 ± 9.3
**Sex,% (*n*)**			
Male	30 (3)	30 (3)	30 (6)
Female	70 (7)	70 (7)	70 (14)
**Race/ethnicity,% (*n*)**			
White	90 (9)	90 (9)	90 (18)
Asian	10 (1)	10 (1)	10 (2)
Height (cm)	166.4 ± 12.7	172.8 ± 10.0	169.6 ± 11.6
Weight (kg)	86.9 ± 18.5	99.4 ± 25.6	93.2 ± 22.7
Body fat (%)	38.2 ± 7.4	42.0 ± 8.2	40.1 ± 7.8
Body mass index (kg/m^2^)	31.3 ± 5.1	33.6 ± 9.7	32.4 ± 7.6
Waist circumference (cm)	98.0 ± 9.8	108.9 ± 17.8	103.4 ± 15.1
Physical Activity (kcals/day)	322 ± 274	289 ± 188	305.6 ± 229.9
**Dietary intake**			
Kcals	2452 ± 526	2483 ± 473	2467 ± 487
Carbohydrates (g)	256.9 ± 75.5	268.3 ± 82.4	262.7 ± 77.1
Sugar (g)	99.6 ± 54.6	106.9 ± 46.1	103.3 ± 49.4
Fiber (g)	20.1 ± 8.9	19.3 ± 8.0	19.6 ± 8.3
Protein (g)	93.7 ± 25.3	105.3 ± 33.4	99.5 ± 29.5
Fat (g)	103.9 ± 26.2	109.6 ± 30.6	106.8 ± 27.8
Sodium (mg)	3456.5 ± 1088.9	3196.9 ± 1392.8	3326.7 ± 1224.1

Reported as mean ± SD unless stated otherwise.

### Gastrointestinal symptoms reduced in IF2-P participants

A baseline assessment of individual GSRS scores revealed no difference between IF1-P and IF2-P (*p* > 0.05; Supplementary Table 1). Upon summing GSRS scores, 39% of IF1-P and 45% of IF2-P participants reported at least one symptom (score ≥ 2) for total GI symptoms, and 17% of IF1 and 12% of IF2-P reported at least one moderate symptom (score ≥ 4; Table 2). There were no significant differences between groups at baseline for any GI symptom clusters (*p* ≥ 0.567). However, after comparing baseline to post-intervention symptom prevalence, significant reductions in total and moderate GI symptom presence were found in IF2-P (*p* < 0.001 and *p* = 0.017, respectively). Similar findings were found in IF2-P for reductions in total upper and lower symptom presence (*p* = 0.031 and *p* = 0.013, respectfully), though no significant reductions were noted for moderate upper and lower symptom presence (*p* ≥ 0.146). In comparison, there were no significant changes for total or moderate upper, lower, and overall symptom presence in IF1-P (*p* ≥ 0.125). For stool characteristics, there were no significant changes from baseline or between groups for stool weight, BSS, or stool pH (*p* ≥ 0.146; Table 2). Stool weights were categorized as low for Western populations (i.e., 80–120 g/day) (61), whereas values for BSS were generally within the range of an ideal stool, indicating normal colonic transit time and ease of defecating while not containing excess liquid. Stool pH was within a healthy range (reference range: 6.5–7.5). Similarly, detected concentrations of SCFAs, including acetate, propionate, isobutyrate, and butyrate, were within normal ranges but did not differ significantly by time or interaction (*p* ≥ 0.126; Supplementary Table 2).

**TABLE 2:** Self-reported gastrointestinal (GI) symptoms and stool characteristics between IF1-P and IF2-P at baseline and week 4.

Variable	Baseline		Week 4	
	IF1-P (*n* = 10)	IF2-P (*n* = 10)	IF1-P (*n* = 10)	IF2-P (*n* = 10)
Total GI scores ≥ 2	43 (39%)	49 (45%)	37 (34%)	30 (27%)*
Total GI scores ≥ 4	19 (17%)	13 (12%)	8 (7%)	4 (4%)*
Total upper GI scores ≥ 2	21 (35%)	26 (43%)	16 (27%)	17 (28%)*
Total upper GI scores ≥ 4	12 (20%)	3 (5%)	1 (2%)	0 (0%)
Total lower GI scores ≥ 2	22 (44%)	23 (38%)	21 (35%)	13 (22%)*
Total lower GI scores ≥ 4	7 (14%)	10 (20%)	7 (14%)	4 (8%)
Stool weight (g)	103.20 ± 92.65	78.01 ± 40.31	65.98 ± 30.11	106.33 ± 64.49
BSS	3.50 ± 1.08	3.20 ± 1.39	2.80 ± 1.31	3.30 ± 1.41
Stool pH	6.97 ± 0.81	6.94 ± 0.32	6.76 ± 0.34	6.95 ± 0.35

GI scores are displayed as the sum of GI symptoms and the percent of participants reporting ≥ 1 symptom per category. Stool characteristic data are reported as mean ± SD. *Significant decrease from baseline values, *p* < 0.05.

### Structure of the gut microbiome altered after short-term fasting

**FIGURE 2:**
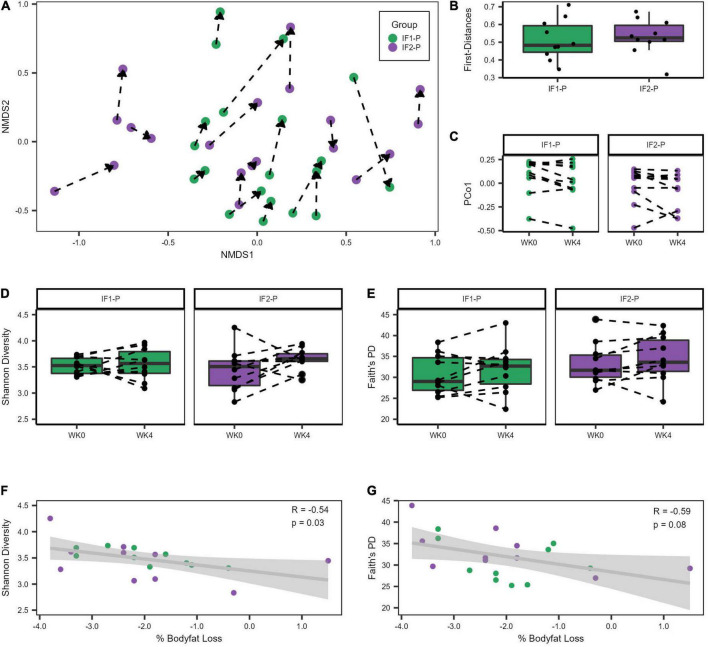
Variation in gut microbiome diversity metrics at baseline and week four of IF1-P and IF2-P participants. **(A)** Non-metric multidimensional scaling (NMDS) ordination of Bray-Curtis dissimilarity matrix. The GM of both IF1-P (*n* = 10) and IF2-P (*n* = 10) groups shifted significantly from baseline to week four (*R*^2^ = 0.042, *p* < 0.001), but there was no difference between groups by time (*R*^2^ = 0.009, *p* = 0.823). The same participant is connected by a dotted line, starting at baseline and the arrow pointing to the end of the intervention period. **(B)** The first distances of the Bray-Curtis dissimilarities between IF1-P and IF2-P were not significant (*p* = 0.579). Boxes denote the interquartile range (IQR) between the first and third quartiles, and the horizontal line defines the median. **(C)** First principal coordinate (PCo1) values differed by time (*p* = 0.013), with no differences between groups over time detected (*p* = 0.473). **(D)** Shannon diversity index did not change significantly over time for IF1-P and IF2-P groups (*p* ≥ 0.341). **(E)** Faith’s PD diversity index did not change significantly over time for IF1-P and IF2-P groups (*p* = 0.653). Boxes denote the IQR between the first and third quartiles, and the horizontal line defines the median. A dotted line connects the same participant. **(F)** Correlation of baseline Shannon Diversity with percent body fat loss from the four-week IF intervention. **(G)** Correlation of baseline Faith’s phylogenetic diversity (PD) with percent body fat loss from the four-week IF intervention. Both groups were combined for the correlation analyses and are displayed in different colors. The gray cloud around the regression line indicates the 95% confidence interval.

Both IF groups’ baseline microbial community structures were significantly altered after the four-week dietary intervention as assessed by the Bray-Curtis beta diversity metric and visualized by non-metric multidimensional scaling (NMDS) ordination (*R*^2^ = 0.042; *p* < 0.001; Figure 2A; Supplementary Table 3), though no significant differences between IF1-P and IF2-P were noted (*R*^2^ = 0.009, *p* = 0.823). Homogeneity of dispersion tests did not reveal any significant differences (*p* ≥ 0.191), increasing our confidence that the significant compositional differences were not an artifact of variance in group dispersion. Comparison of the first distances of Bray-Curtis dissimilarity between groups was non-significant (*p* = 0.579; Figure 2B), indicating no difference in the change in dissimilarity from baseline between IF1-P and IF2-P. Results from the PERMANOVA analysis were paralleled with the LME model, which identified the overall IF-P intervention as a significant factor in the participant’s GM composition (time effect, *p* = 0.013), with no differences between groups detected (group × time effect, *p* = 0.473; Figure 2C). Null findings were observed for Shannon diversity and Faith’s PD, with no significant effects for time (*p* ≥ 0.155) or interaction (*p* ≥ 0.341; Figures 2D,E). Overall, these data show alpha diversity was unaffected by this short-term intervention. However, there was a significant negative correlation between baseline Shannon diversity and percent body fat change after the four-week intervention (*R*^2^ = 0.287, *p* = 0.030; Figure 2F). Therefore, individuals with increased baseline Shannon diversity had the greatest reduction in body fat percentage. This finding was not paralleled with Faith’s PD, though it was trending toward significance (*R*^2^ = 0.358, *p* = 0.084; Figure 2G).

### Alterations in gut microbiome composition and predicted function

**FIGURE 3:**
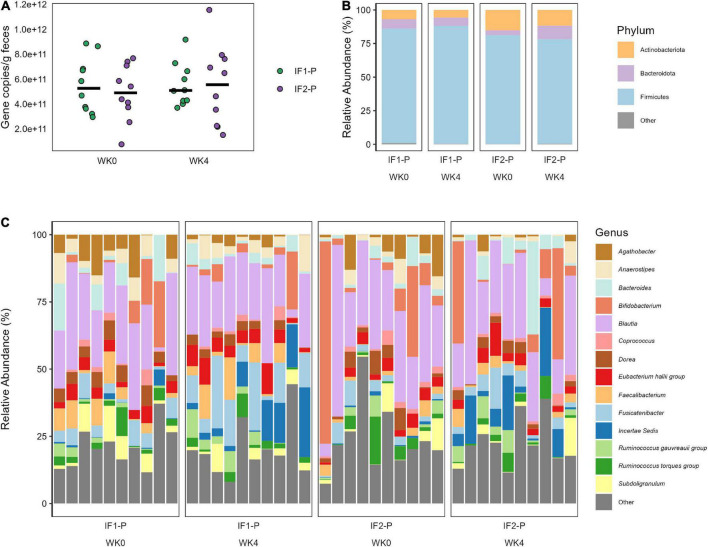
**(A)** Total bacterial number observed in both IF1-P (*n* = 10) and IF2-P (*n* = 10) groups at baseline and week four. No significant effects of time or interaction (*p* ≥ 0.603) were noted. Total bacterial numbers were calculated as average copies of 16S rRNA gene/g wet feces via qPCR. Group means at each time point are displayed as black bars. **(B)** Average relative abundance of the most prevalent gut microbiome phyla among study participants for IF1-P (*n* = 10) and IF2-P (*n* = 10) at baseline and week four by 16S rRNA sequencing. Phyla with a median relative abundance of less than 1% are collapsed into the category “Other”. **(C)** Average relative abundance of the most prevalent gut microbiome genera among study participants for IF1-P (*n* = 10) and IF2-P (*n* = 10) at baseline and week four by 16S rRNA sequencing. Genera with a median relative abundance of less than 1% are collapsed into the category “Other”.

For the qPCR analysis, no significant time (*p* = 0.603) or interaction effects (*p* = 0.653) in total bacterial numbers were detected, indicating the estimated number of microbes remained relatively constant throughout the study and across groups (Figure 3A). With high throughput 16S amplicon sequencing, we identified 115 ASVs after filtering, represented by five phyla and 67 genera (Class: 8; Order: 17; Family: 26). At baseline, the composition of the gut microbiome for both groups was dominated by Firmicutes (IF1-P: 85.1% vs. IF2-P: 81.1%), followed by Actinobacteriota (6.9% vs. 15.2%), Bacteroidota (7.2% vs. 3.5%), Proteobacteria (0.8% vs. 0.2%), and Desulfobacterota (0.1% vs. < 0.1%). The GM at the genus level displayed much greater variation by individual. No taxa above the genus level differed significantly in their abundances from baseline to week four, nor was there a significant time or interaction effect for the Firmicutes/Bacteroidota ratio (*p* ≥ 0.527). The abundances of phyla and genera (at an individual level) at baseline and week four for each group with greater than 1% mean relative abundance are shown in Figures 3B,C, respectfully.

**FIGURE 4:**
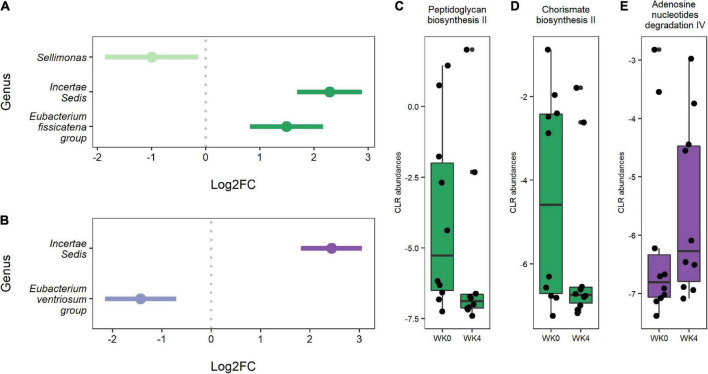
**(A)** Genera were differentially abundant between baseline and week four for IF1-P participants (*n* = 10). **(B)** Genera that were differentially abundant between baseline and week four for IF2-P participants (*n* = 10). Points represent each genera’s log2 fold change (log2FC; effect size). A positive value indicates that a feature increased in abundance at week four (right), and a negative value indicates a decrease in abundance at week four (left). Bars represent 95% confidence intervals derived from the ANCOM-BC model. Note, the genus *Incertae Sedis* is from the Ruminococcaceae family. Boxplots displaying statistically significant differences in **(C)** peptidoglycan biosynthesis II and **(D)** chorismate biosynthesis II from baseline to week four of predicted metabolic pathways for IF1-P participants. Boxplot displaying a statistically significant difference in **(E)** adenosine nucleotides degradation IV from baseline to week four of the predicted metabolic pathway for IF2-P participants. Pathways are displayed as centered log-ratio (CLR) transformed abundances. Boxes denote the interquartile range (IQR) between the first and third quartiles, and the horizontal line defines the median.

Differential abundance testing identified three genera that significantly changed from baseline to week four in the IF1-P group, including a decrease in *Sellimonas* (log2FC = −0.997, *q* < 0.001) and an increase in Ruminococcaceae *Incertae Sedis* (log2FC = 2.289, *q* = 0.007) and *Eubacterium fissicatena group* (log2FC = 2.215, *q* < 0.001; Figure 4A). In comparison, two genera significantly changed in the IF2-P group, including an increase in Ruminococcaceae *Incertae Sedis* (log2FC = 2.435, *q* = 0.005) and a decrease in *Eubacterium ventriosum group* (log2FC = −1.990, *q* = 0.001) (Figure 4B). Analysis between the change in these differently abundance genera with change in percent body did not reveal any significant associations, though *Sellimonas* was trending toward a negative correlation (*R*^2^ = −0.274, *q* = 0.072). Predicted functional composition of microbial communities was assessed via PICRUSt2, identifying 265 MetaCyc pathways after filtering. Upon differential abundance testing, IF1-P had a significant decrease in the predicted function of peptidoglycan biosynthesis II (log2FC = −1.674; *q* < 0.001) and chorismate biosynthesis II (log2FC = −1.509, *q* < 0.001), whereas IF2-P had a significant increase in the predicted function of adenosine nucleotides degradation IV (log2FC = 0.719, *q* < 0.001; Figures 4C–E).

### Alterations in the plasma metabolome between groups after short-term fasting

A total of 138 aqueous metabolites were reliably detected across 40 samples (i.e., QC CV < 20% and relative abundance > 1,000 in 80% of samples). Data were log_10_-transformed, and Pareto scaled (mean-centered and divided by the square root of the standard deviation of each variable) prior to all subsequent analyses and visualizations (Supplementary Figure 1). Following normalization, Levene’s test of homogeneity showed equal variance between study groups (*p* > 0.05). Outlier analysis was performed via random forest (RF) and principal component analysis (PCA). RF performed with 500 decision trees indicated five potential outliers according to the greatest outlying measure (Supplementary Figure 2A), although no sample fell outside of the 95% CI as indicated by two-dimensional PCA (Supplementary Figure 2B). As such, no samples were confirmed as outliers, and all data were retained for subsequent analysis.

**FIGURE 5:**
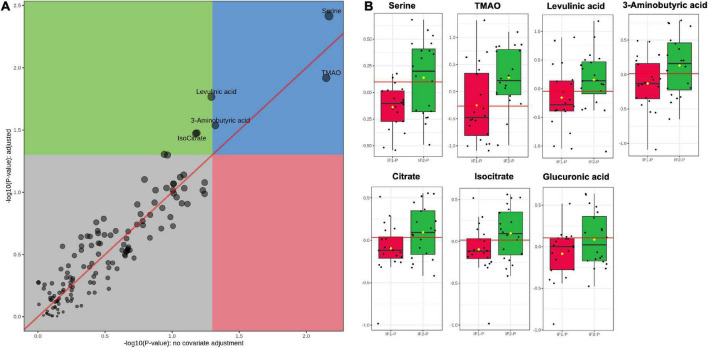
**(A)** GLM adjusted for age, sex, BMI, and time, with FDR-correction, and **(B)** box plots of significant metabolites as indicated by GLM: serine (*q* = 0.003), TMAO (*q* = 0.012), levulinic acid (*q* = 0.017), 3-aminobutyric acid (*q* = 0.029), citrate (*q* = 0.033), isocitrate (*q* = 0.033), and glucuronic acid (*q* = 0.049). Red lines in box plots denote optimal cutoff values as calculated by the Youden method, black lines indicate median values, and yellow diamonds show group averages.

Next, we performed ANOVA-simultaneous component analysis (ASCA) to determine the significant main effects of time and group interactions. As can be seen in Supplementary Figure 3A, little separation was observed in the three-dimensional PCA, showing no observable clustering due to time or group. However, the ASCA did reveal a significant effect of time (*p* < 0.01; Supplementary Figure 3B), but no significant effect of group (*p* = 0.17, Supplementary Figure 3C) or any significant interactions between time and group (*p* = 0.78; Supplementary Figure 3D). Given the significant effect of time and our interest in the effects of the IF-P group, we prepared a GLM with age, sex, BMI, and time as covariates and corrected for FDR (Figure 5A). When controlling for these relevant covariates, we observed significant effects of IF-P on seven metabolites (Figure 5B, Supplementary Table 4), which may be considered candidate markers of intermittent fasting: serine (*q* = 0.003), TMAO (*q* = 0.012), levulinic acid (*q* = 0.017), 3-aminobutyric acid (*q* = 0.029), citrate (*q* = 0.033), isocitrate (*q* = 0.033), and glucuronic acid (*q* = 0.049).

Fold change (FC) and receiver operating characteristic (ROC) analyses assessed the magnitude of change between IF-P groups and the univariate classification performance of the candidate fasting markers, respectively. For FC analysis, groups were analyzed as IF2-P/IF1-P. While all seven significant metabolites were increased in the IF2-P group, the magnitude of change was consistent; FC ranged from 1.14 to 1.60 (see Supplementary Table 4 for significance, FC, and AUC details of metabolite markers). The area under the curve (AUC) estimates for individual candidate markers ranged from 0.63 to 0.74. Although no significant correlations were observed between changes in metabolite levels and percent body fat, positive associations were observed between citrate and glucuronic acid (*R*^2^ = 0.282, *p* = 0.019), glucuronic acid and 3-aminobutyric acid (*R*^2^ = 0.311, *p* = 0.013), and between 3-aminobutyric acid and serine (*R*^2^ = 0.271, *p* = 0.022).

**FIGURE 6:**
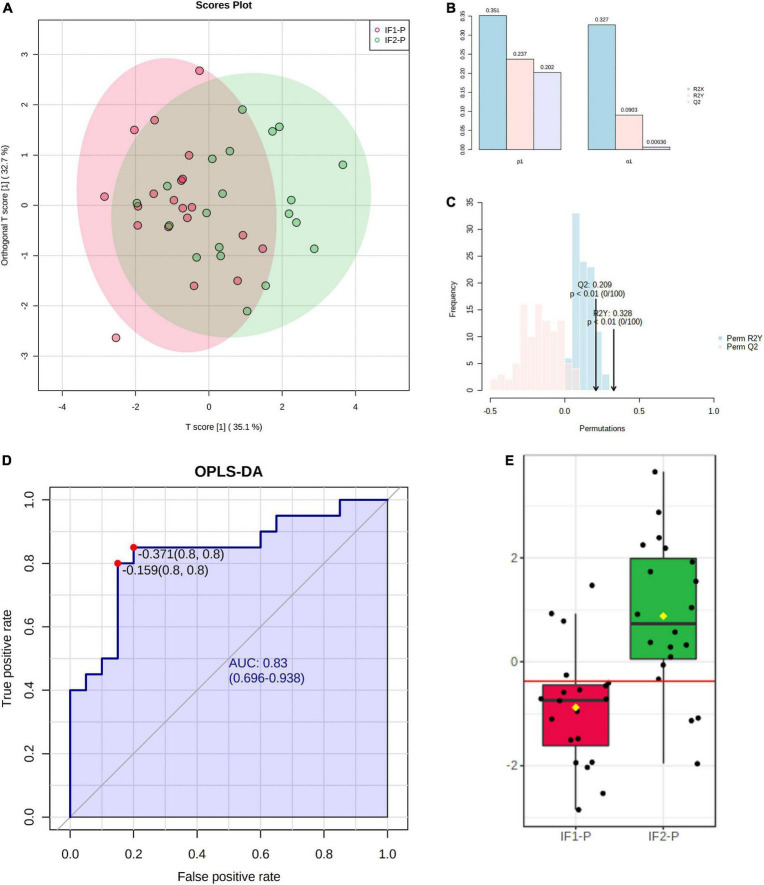
**(A)** Scores plot of OPLS-DA model constructed using seven significant metabolites identified by GLM, showing percent variance accounted for by experimental and orthogonal data. **(B)** OPLS-DA model overview showing predictive and explanatory capacity (*R^2^X* = 0.351, *R^2^Y* = 0.237, *Q^2^* = 0.202); *y*-axis represents proportion of total variance. **(C)** Permutation test with 100 iterations showing model fit distributions (Perm *R^2^Y p* < 0.01, Perm *Q^2^ p* < 0.01). **(D)** ROC analysis of OPLS-DA model for assessing IF-P (AUC = 0.83, 95% CI: 0.70-0.94, sensitivity = 0.8, specificity = 0.8). **(E)** Box plot of OPLS-DA predictive values; the red line in the box plot denotes the optimal cutoff value, while yellow diamonds show group averages, and black lines illustrate group medians.

To increase the predictive accuracy of the candidate marker panel, we constructed a multivariate orthogonal partial least squares-discriminant analysis (OPLS-DA) model using the seven significant metabolites identified by GLM. Analysis of the OPLS-DA scores plot revealed the model to account for more variance than an orthogonal data matrix of equal dimension (Figure 6A). While the OPLS-DA model showed low-to-moderate predictive and explanatory capacity (*R^2^X* = 0.351, *R^2^Y* = 0.237, *Q^2^* = 0.202) (Figure 6B), permutation testing showed good fit to data and, notably, did not indicate model overfitting (Perm *R^2^Y p* < 0.01, Perm *Q^2^ p* < 0.01) (Figure 6C). Following model construction and validation, we performed ROC analysis to assess the classification performance of the multivariate OPLS-DA model. ROC analysis showed good accuracy of the model for assessing the duration of the IF-P intervention; AUC was observed to be 0.83 (95% CI: 0.70-0.94) when sensitivity and specificity were set to 0.80 (Figure 6D). Importantly, the OPLS-DA model provided greater classification accuracy than any significant metabolite individually. A box plot of model-implied *Y*-values derived from the OPLS-DA model is visualized between IF1-P and IF2-P groups in Figure 6E.

Next, we performed debiased sparse partial correlation (DSPC) networking modeling and metabolite ontology analysis to evaluate pathway-informed correlations and metabolite localization, respectively. The DSPC analysis was based on a least absolute shrinkage and selective operator (LASSO) algorithm, and revealed significant functional correlations in valine, leucine, and isoleucine biosynthesis (*p* = 8.59E-9) and degradation (*p* = 7.91E-5), aminoacyl-tRNA biosynthesis (*p* = 1.94E-4), fatty acid biosynthesis (*p* = 0.018), and phenylalanine, tyrosine, and tryptophan biosynthesis (*p* = 0.048) (Supplementary Figure 4). Qualitative metabolite ontology analysis was performed to identify organ, tissue, cellular, and subcellular localizations of study metabolites. Although metabolome coverage is admittedly limited given our targeted MS approach, network visualization of ontology results showed our experimental metabolites were derived, in order of most to least, from the central nervous system, brain, neuron, prostate, and mitochondria (Supplementary Figure 5).

## Discussion

The GM is tightly intertwined with host health, which has raised considerable interest in how the resident GI microbes respond to dietary intervention. Advances in understanding how the GM influences and is modified by body weight will allow for a greater understanding of this relationship. Moreover, changes in the GM and associated metabolites may provide optimal dietary input for GM modulation and, ultimately, host health. Therefore, in this exploratory analysis, we sought to examine the GM and plasma metabolome of a subgroup of adult participants with overweight/obesity who were randomized into an IF-P regimen of one versus two days. Over four weeks, participants in both groups lost a significant amount of body fat with associated metabolic improvements. In contrast, the community structure of the GM was minimally impacted, with beta-diversity shifting approximately 4%. Similarly, differential abundance analysis revealed some shifts at the genus level, including increased *Incertae sedis* (from the Ruminococcaceae family) abundance in both groups. Analysis of the plasma metabolome revealed a significant increase in seven out of 138 validated metabolites in the IF2-P group. Overall, these results support that controlled, short-term IF-P regimens modestly impact the GM. Perhaps more importantly, the GM displayed resiliency and marked inter-individuality despite the significant weight loss and cardiometabolic improvement in these overweight/obese participants.

To our knowledge, this study is the first to directly compare two IF regimens on longitudinal GM changes. While a direct comparison to similar work is limited, our results generally align with the results from short-term inventions. For example, in an energy-restricted IF intervention using a modified fasting regimen of 5:2 (5 days normal feeding:2 days fasting), 12 weeks of energy restriction elicited approximately a 5% reduction in weight (62). While weight loss was associated with significant metabolic improvements, comparatively, there was less change in the GM and targeted plasma metabolome. However, the GM composition at baseline was moderately predictive of weight loss. Similarly, we noted baseline alpha diversity was significantly correlated with loss in percentage body fat, suggesting that the initial community dynamics of the GM may be an important determinant in response to periods of fasting and nutritional interventions. These findings add to previous reports that the GM of participants entering weight-loss interventions may greatly influence the host’s response to these dietary regimens (63, 64). In terms of the baseline profile of the GM, increased diversity has been associated with enhanced response to a dietary weight loss intervention (65). Other calorie restriction interventions have noted increased alpha diversity (Shannon index) over short-term periods by implementing a high protein diet (30% of daily kcal) (21). Participants in the current study consumed 35% of their total energy intake from protein, though we only reported results after four weeks, whereas the other study had a longer intervention (eight weeks). In another high-protein CR intervention, protein supplementation had little effect on microbial diversity and relative abundance (22). However, decreased body weight and fat mass were significantly correlated with increased microbial diversity.

Differential abundance analysis revealed increases in several genera, including *Incertae sedis* and *Eubacterium fissicatena group*, and a decrease in *Sellimonas* in IF1-P. *Incertae sedis* is an unclassified genus from the Ruminococceacae family, a group of strict anaerobes normally present in healthy individuals (66). Overall, the family Ruminococcaceae plays an important role in maintaining gut health through the potential to produce butyrate and other SCFAs. *Eubacterium* is phylogenetically diverse and forms the core genera of health-associated human GM (67). Indeed, multiple species of this genus are currently regarded as promising targets for microbial therapeutics (67), though *Eubacterium fissicatena group* remains a poorly described genus, with few clinical studies detecting its presence (68). *Sellimonas* is also a genus less characterized. Under the Lachnospiraceae family, *Sellimonas* contains certain species that have been proposed as an indicator of GI homeostasis (69). *Incertae sedis* was also increased in IF2-P, while *Eubacterium ventriosum group* decreased. The decrease in *Eubacterium ventriosum group* was of interest as this microbe was previously found to be enriched in high vs. low-BMI twin-dyads (70). Similar findings have been reported in a broader examination of unrelated obese vs. non-obese study participants (71). It may be that these butyrate producers provide substrate to the host as energy harvesters, which could contribute to a positive energy balance. Conversely, reduced abundance of this genus is well noted in colorectal cancer patients (72–75) and has been proposed as a biomarker for lower disease risk (67). Such findings display the complexity of *Eubacterium*, highlighting the importance of better mapping function and host-health implications at the genus and species level.

Less affected were the predicted functional pathways of the GM. IF1-P resulted in a significant decrease in two predicted functions. This included peptidoglycan biosynthesis II, which is a step in forming cell wall components of Gram-positive bacteria, including pathogenic species (like *Staphylococcus aureus*), and plays a role in GM-derived inflammation in the host (76). Previously, IF in a diabetic mice model reduced plasma peptidoglycan, a surrogate marker for gut barrier function (77). The other pathway, chorismate biosynthesis II, contributes to chorismate synthesis and is a precursor to tryptophan and subsequent serotonin production in the GI tract. IF2-P resulted in a significant increase in adenosine nucleotides degradation IV, which may signal increased microbe and energy turnover.

Regarding the plasma metabolome, we identified an increase in several metabolites that were significant over time and increased in the IF2-P group. The elevation in these specific metabolites was intriguing as some are linked to healthy states, while others are linked to disease. For example, serine plays an important role in glutathione production (78), citrate and its isomer, isocitrate, are involved in energy metabolism, and glucuronic acid is involved in detoxifying xenobiotic compounds and has been proposed as a biomarker of longevity (79). In contrast, TMAO is a compound found in high protein foods (e.g., fish) and can be generated from precursors like choline and carnitine (e.g., found in eggs and beef) and has been associated with heart disease (80, 81). Concentrations of TMAO in circulation appear reliant on dietary intake (82, 83). Like participants in the present study, healthy individuals with a high relative abundance of Firmicutes have been reported to have greater circulating levels of TMAO (82). While increased levels of this metabolite have been associated with metabolic disease, participants in the present study displayed significant improvements in cardiometabolic outcomes, including lipid profiles. In relation to diet, a notable component of both the IF1-P and IF2-P diet was resistant starch, which has been reported to be associated with higher circulating TMAO levels when overall carbohydrates are reduced, and protein levels increased (84). We have used a similar dietary intervention, noting substantial improvements in postprandial metabolism, including reduced glucose and insulin response (85, 86). Therefore, it may be that circulating concentrations of TMAO were reflective of differences in the GM community and dietary intake, rather than indicating a role of TMAO in negative cardiometabolic outcomes in the present study. More work exploring the impact of increased resistant starch and relative protein during caloric restriction is warranted, particularly concerning the GM and different metabolomic assessments (e.g., contents of the GI tract at different sections). Relatedly, while fecal metabolites might be more reflective of the direct metabolic output of the microbiota, blood metabolites provide a window into which of these compounds make it into circulation (18, 79). Indeed, the plasma metabolome is connected and partially reflective of the GM as it can present a person-specific signature and is largely predicted by host-associated characteristics (87–89).

Regarding metabolomic pathway analysis, our DSPC model detected several negative correlations in accordance with calorie restriction, including biosynthesis of the amino acids valine, leucine, isoleucine, phenylalanine, tyrosine, and tryptophan. While decreases in plasma branched-chain and aromatic amino acids are reportedly associated with weight loss and improvement in insulin resistance in obese individuals (90, 91), the implications of reduced tryptophan synthesis are less clear. Decreased circulating levels of tryptophan may influence the serotoninergic system and mood. Reductions have been observed in other short-term weight loss trials with suggestive susceptibility to food cravings (92). In contrast, these behaviors were not reflected in participants from the present study as hunger ratings significantly improved over baseline levels (31). The other notable negative correlation was fatty acid biosynthesis. Interestingly, the current study showed significant loss of body weight, total and abdominal fat loss, despite not detecting a significant association with fat oxidation. Although, alterations in fatty acid synthesis have displayed a greater magnitude of change over fatty acid oxidation in preclinical models of calorie restriction and appears to be an important metabolic adaptation to reduced energy states (93, 94). In particular, decreased saturated fatty acid synthesis such as palmitic acid, myristic acid, and capric acid appears to be associated with cell longevity and protective against cancerous traits, perhaps mediated through the *p53* tumor suppressor gene (95). These results are notable considering the short-term intervention period and warrant future exploration with expanded omic surveys and longer time durations.

Finally, we captured self-reported GI symptoms and stool characteristics (pH, SCFAs, and biomass estimate). While routine, these assessments are generally lacking in diet-focused GM research, and adverse GI symptoms remain highly reported in Western populations (96, 97). We noted a similar trend in the participants in the present study, with nearly half reporting GI symptom presence at baseline. After the four-week intervention, participants in the IF2-P group reported significantly decreased incidence of minor and moderate symptoms, while IF1-P did not. These findings are noteworthy as restrictive diets have been positively associated with self-reported GI issues (98). Differences may have been due to the longer periods of supported fasting, providing “gut rest” (24). We did not find significant changes in stool pH after IF, which is interesting because prolonged fasting (+18 h) has been reported to produce a higher gut pH than constant feeding (99). In relation, SCFA concentrations did not change significantly despite the significant reduction in energy and periods of IF. Presumably, during periods of dietary restraint, the host would more readily absorb these SCFA, resulting in decreased detection in the stool (100). While speculatory, this presumed reduction in SCFAs was bolstered by the significant increase in dietary fiber by approximately 10 grams in the dietary intervention. Much of this increase was supplied by isomaltooligosaccharide, a non-digestible fiber metabolized by the GM (101). Regardless, SCFAs concentrations were within range of those found in stool samples of healthy humans (102, 103). Overall, our findings support the notion that short-term dietary intervention impacts the microbial ecosystem of the human gut, showcasing the resilience of the GM community (104). Indeed, this paralleled the biomass estimates provided by our qPCR analysis, where we did not detect a significant decrease over time.

The present study has several strengths, including a tightly controlled design and well-matched comparator groups regarding participant characteristics and nutrient intake during the intervention. Moreover, we provided a simultaneous investigation of the changes in structure and predicted function of the gut microbiota, plasma metabolome, and host-associated features. However, there are several limitations. First and foremost, the sample size was small. A greater sample and a more robust design protocol, such as a cross-over design, for GM research may better account for interpersonal variabilities. Second, we employed 16S rRNA gene sequencing on fecal samples to assess the GM which constrained our taxonomic resolution and survey of microbial gene content and function. In fasting, where many important microbial changes in more proximal sections of the GI tract are suggested, we were limited by our sample collection. Third, the fecal metabolome was not assessed, which would have better reconciled the apparent gap between the GM and the plasma metabolome. Fourth, we did not collect samples directly after fasting and fed periods to make these important comparisons. Such investigations will likely require time-series and in-patient designs, features that were not within the scope of this current exploratory work. Finally, our participants were overweight/mildly obese individuals with baseline cardiometabolic parameters frequently characterized in the literature as “healthy obese” (31). Generally, host-microbe metabolic associations are more apt to be disrupted in individuals experiencing more severe obesity (i.e., BMI ≥ 35) relative to individuals with a normal weight (BMI < 25) (105). The group BMI means of IF1-P and IF2-P in the current study could be considered only slightly obese (i.e., 31.3 ± 5.1 and 33.6 ± 9.7 kg/m^2^, respectively) and thus may not have been as sensitive to change during the short-term intervention. Indeed, a health-associated GM appears to display resilience to change, including dietary intervention (104). These factors may have contributed to some of the current study’s findings.

## Conclusion

In the current study, we observed that short-term IF-P induced modest shifts in the GM and plasma metabolome, despite conferring significant body weight and fat reduction. These results indicate that IF may promote minor increases in health-associated taxa and alterations in microbial community structure and plasma metabolite profile. However, when controlled for overall energy intake and nutritional profile, fasting for one vs. two days did not promote significant differential changes in this short-term, calorically restricted protein pacing intervention. Importantly, we show that the GM in overweight and obese individuals appears to have great resiliency despite significant energy restriction and fat loss. Moreover, the baseline composition of the GM may be an important variable in weight loss, though larger and longer duration studies are needed to better characterize IF modifications of microbial and metabolic factors.

## Data availability statement

The datasets presented in this study can be found in online repositories. The names of the repository/repositories and accession number(s) can be found below: https://www.ncbi.nlm.nih.gov/, PRJNA847971.

## Ethics statement

The studies involving human participants were reviewed and approved by The Institutional Review Boards of Skidmore College, NY, United States and Arizona State University, AZ, United States. The patients/participants provided their written informed consent to participate in this study.

## Author contributions

AM, EG, KS, and PA: conceptualization. KA, MP, and PA: study implementation and sample collection. AM, PJ, DB, BD, CW, HG, KS, and PA: laboratory methodology and analysis. AM, PJ, and DB: statistical analysis. AM, PJ, DB, EG, KS, and PA: original draft preparation and writing. AM, PJ, DB, BD, CW, KA, MP, HG, EG, KS, and PA: review and editing. AM and PJ: visualization. KS and PA: supervision. All authors contributed to the article and approved the submitted version.
