# Supplementary material for: Exploratory analysis of one versus two-day intermittent fasting protocols on the gut microbiome and plasma metabolome in adults with overweight/obesity
Source: Front Nutr. 2022 Oct 26;9:1036080. doi: 10.3389/fnut.2022.1036080 (PMC9644216; doi:10.3389/fnut.2022.1036080)
Supplement: Supplementary file 1 [file Data_Sheet_1.docx]

Supplementary Material

# Supplementary Figures and Tables

## Supplementary Figures

**
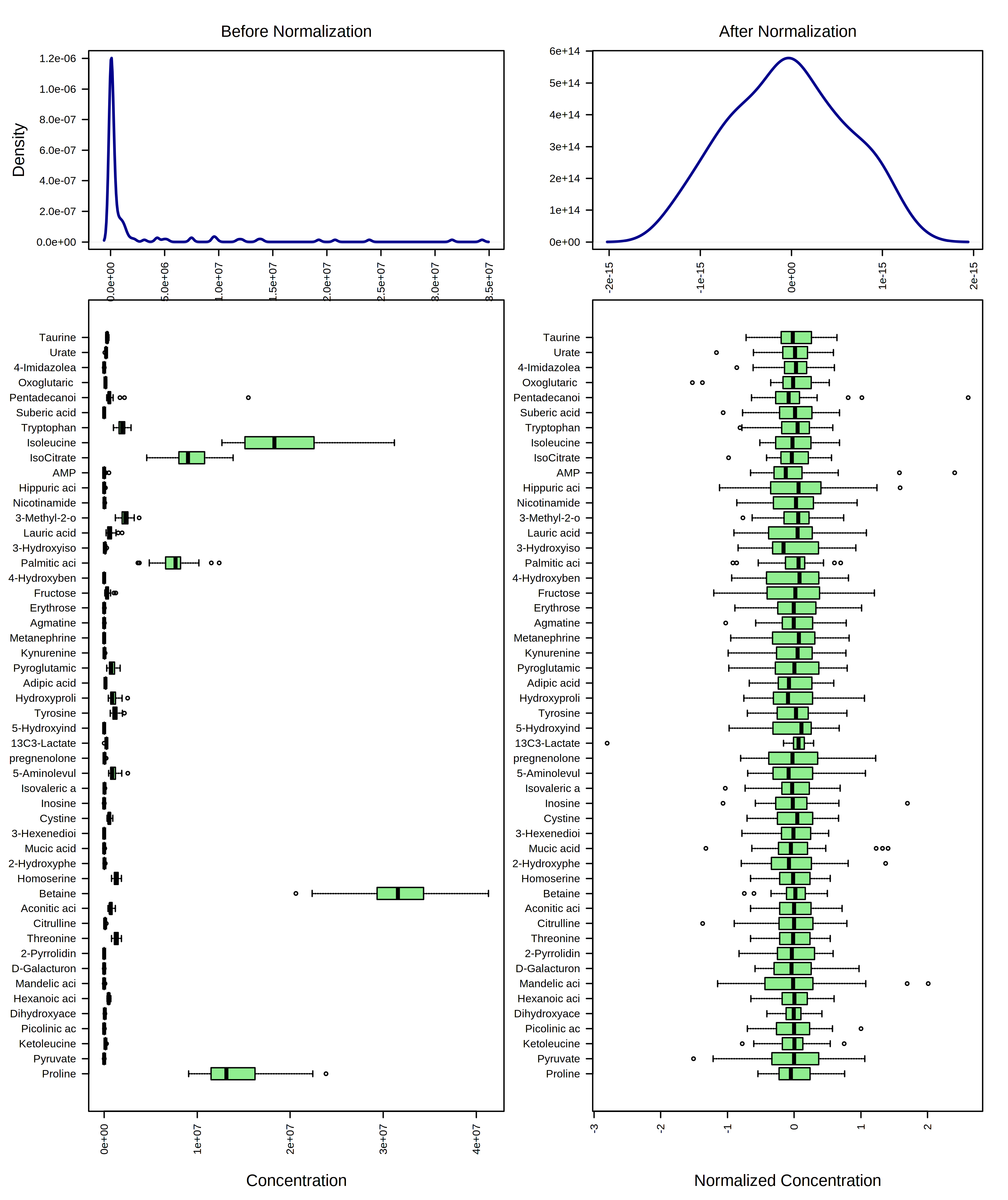
**

**Figure S1.** Distribution of plasma metabolomic data before and after normalization. Data were log_10_-transformed and Pareto scaled (mean-centered and divided by the square root of the standard deviation of each variable) to approximate normality prior to all analyses and visualizations. Levene’s test of homogeneity performed on normalized data showed equal variance (*p* > 0.05).


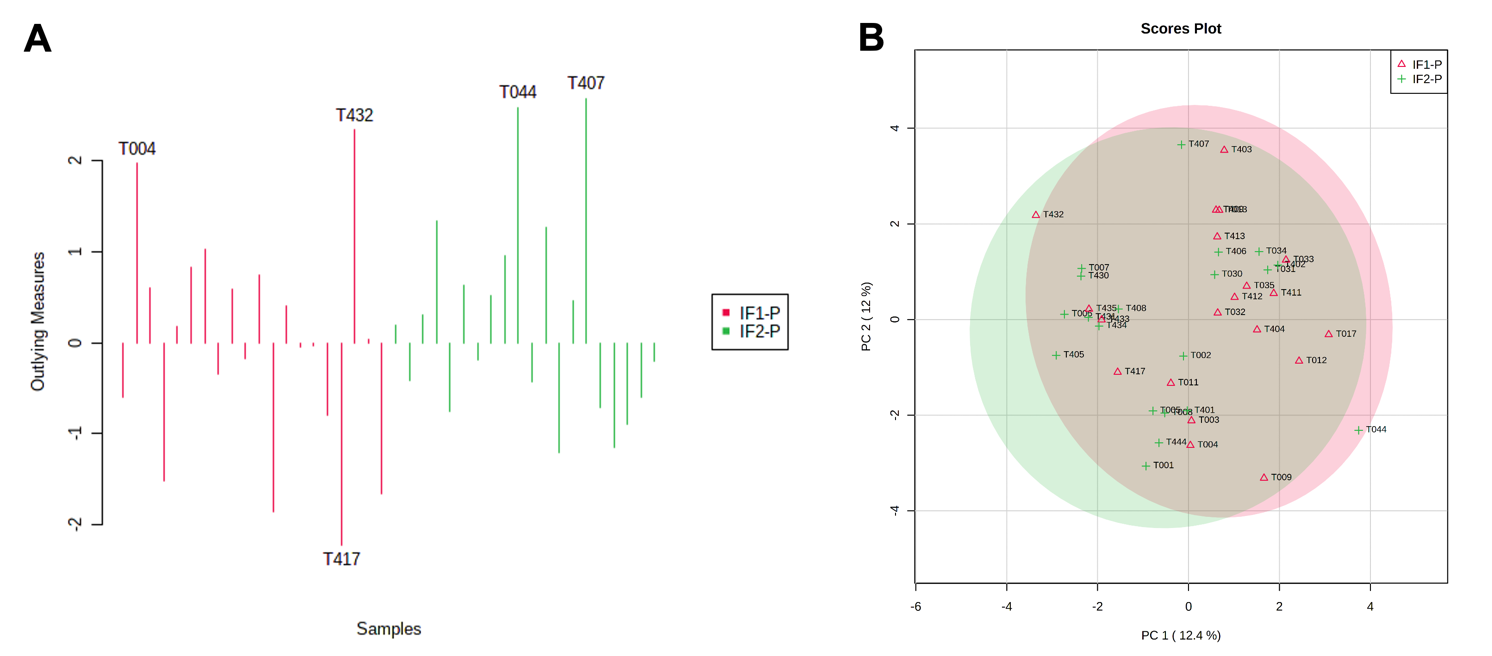


**Figure S2.** Outlier analysis of the plasma metabolome was performed using (**A**) random forest with 500 decision trees, indicating top 5 potential outliers by outlying measures, and (**B**) analysis of 95% confidence intervals of two-dimensional principal components analysis to confirm significant distance from group clusters.


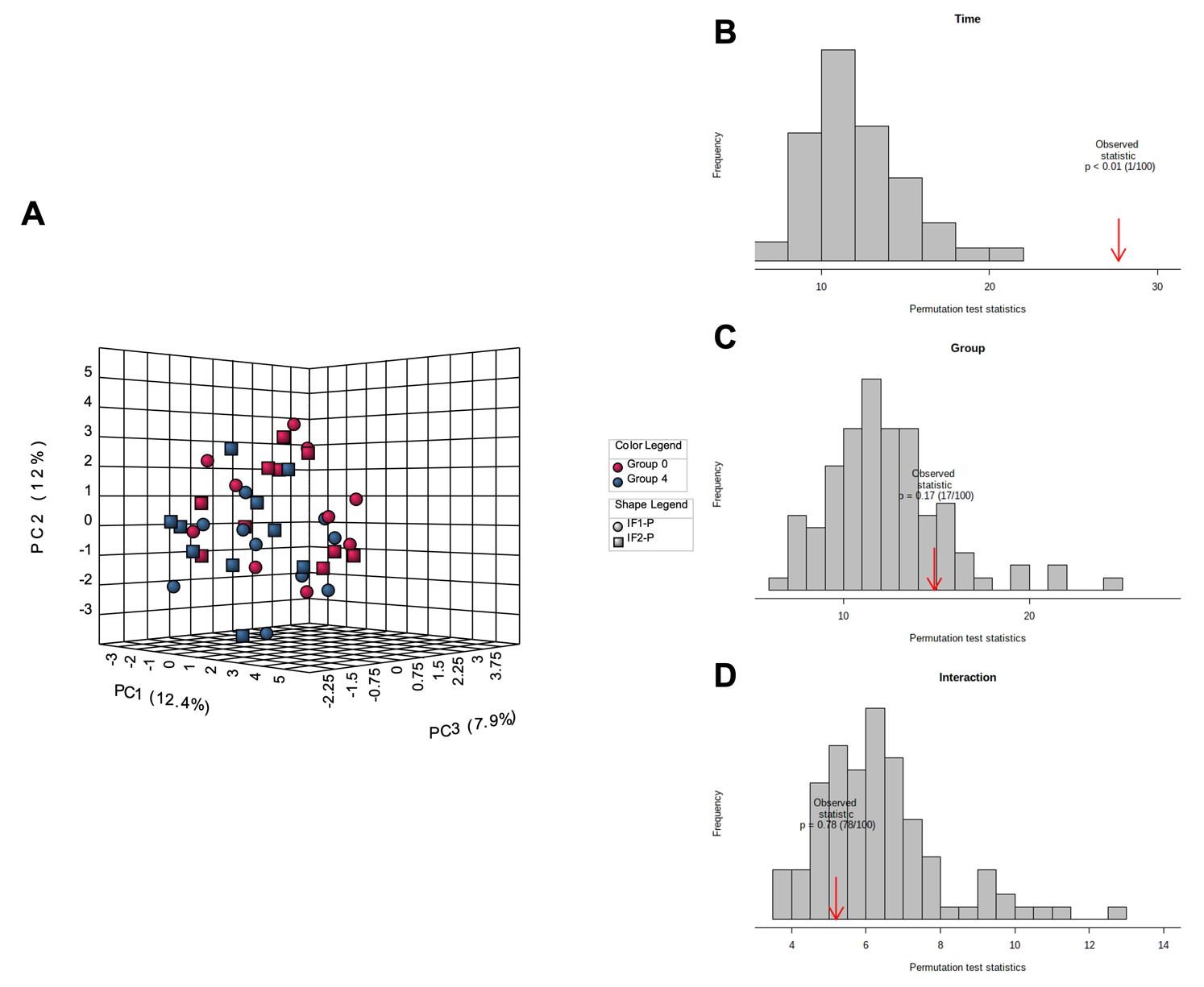


**Figure S3.** Analysis of variance simultaneous-component analysis was performed on all plasma metabolites to test for the main effects of time, group, and interaction effect between terms. (**A**) Three-dimensional, two-factor principal components analysis shows little separation between groups, explaining 32.3% of between-group variance using three principal components, (**B**) distribution of significance by time (*p* < 0.01), (**C**) distribution of significance by group (*p* = 0.17), and (**D**) distribution of significance by time x group interaction (*p* = 0.78). Group 0 and 4 represent baseline and 4-week timepoints.


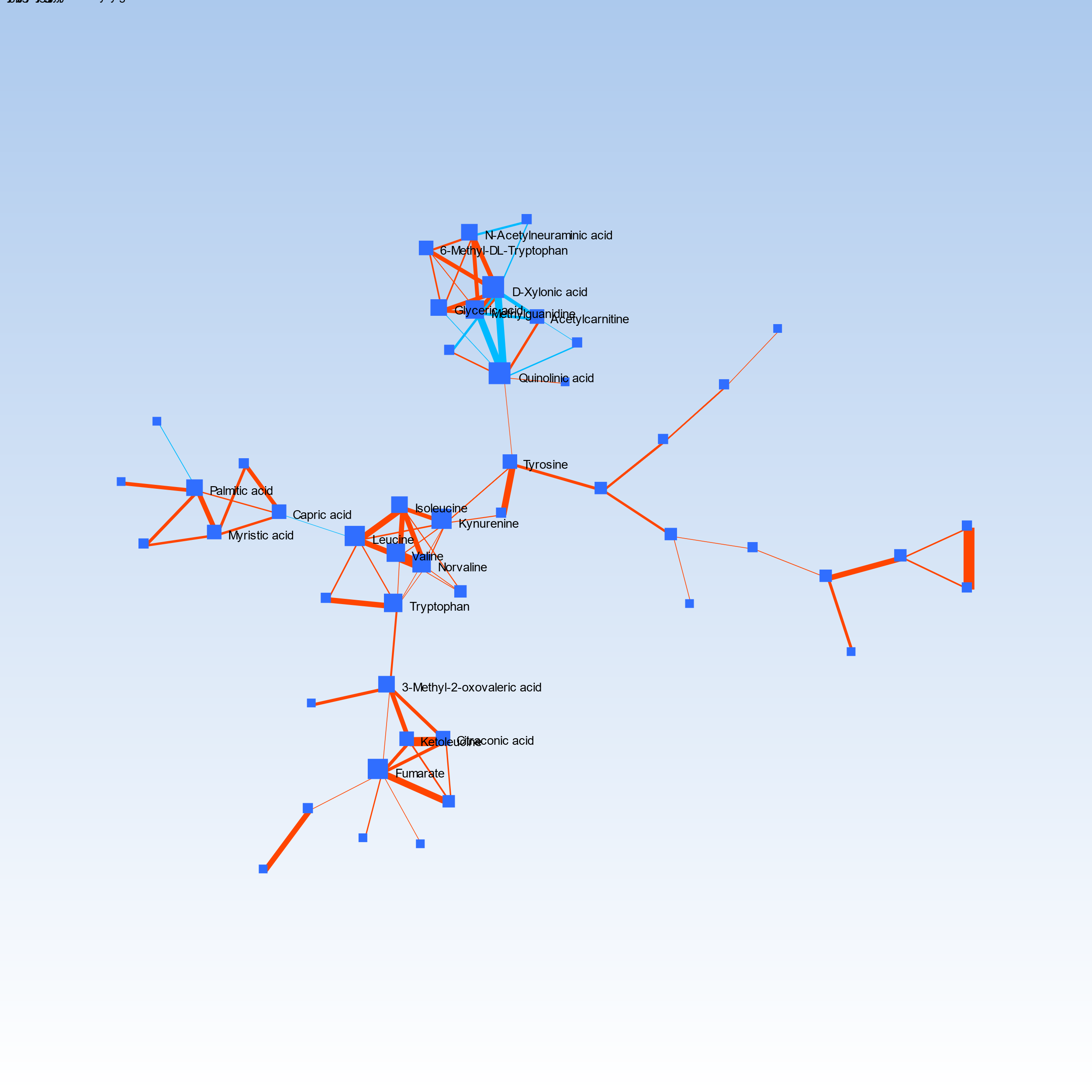


**Figure S4.** Debiased sparse partial correlation network modelling using a least absolute shrinkage and selective operator algorithm shows significant correlations in functional pathways related to valine, leucine and isoleucine biosynthesis (*p* = 8.59E-9) and degradation (*p* = 7.91E-5), aminoacyl-tRNA biosynthesis (*p* = 1.94E-4), fatty acid biosynthesis (*p* = 0.018), and phenylalanine, tyrosine and tryptophan biosynthesis (*p* = 0.048). Nodes denote metabolites, while edges show associations. Positive and negative correlations are visualized in blue and red, respectively; edge width denotes strength of association.


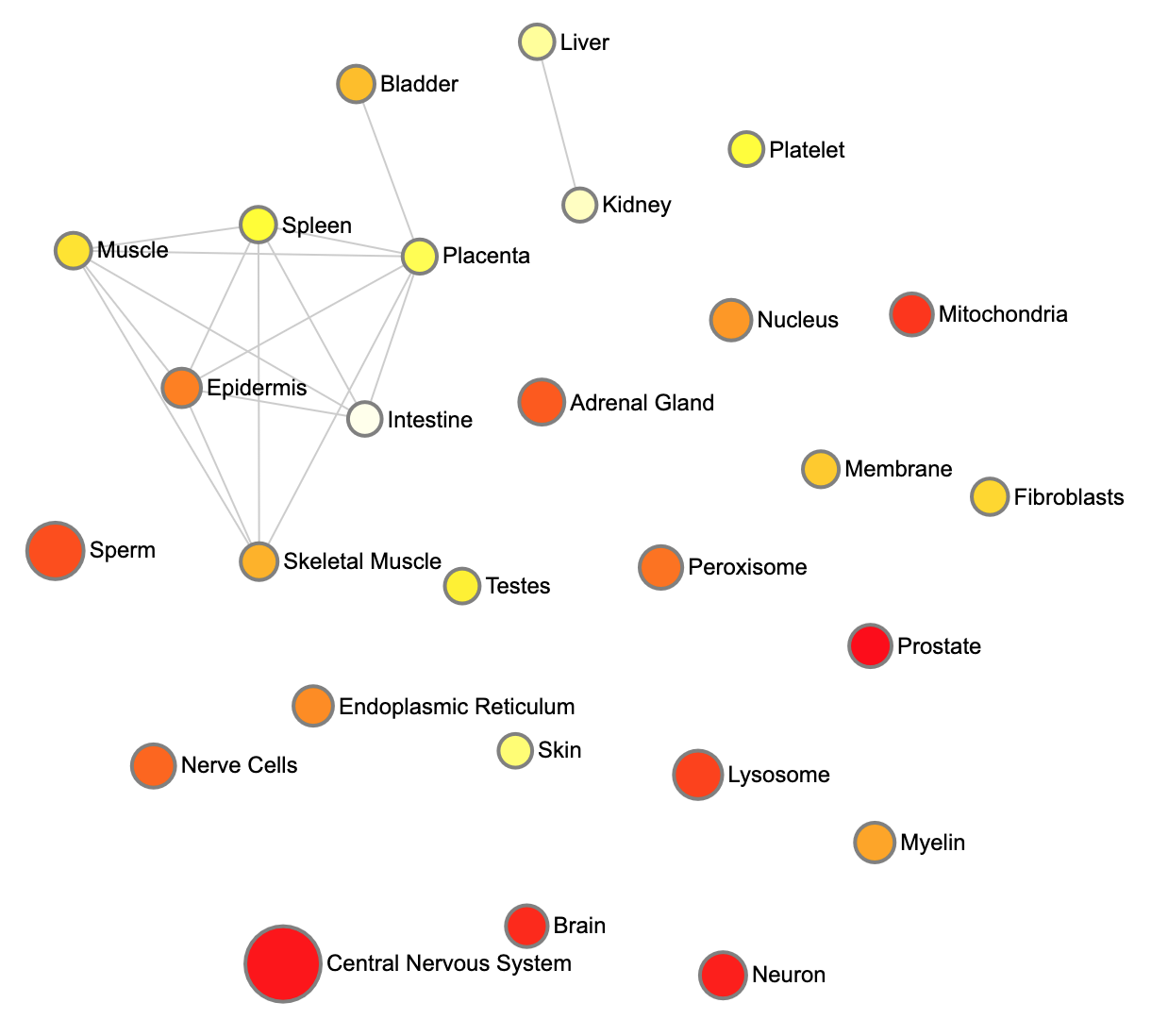


**Figure S5.** Network view of metabolite ontology analysis performed using the total set of reliably detected metabolites from our targeted metabolomic assay (138 aqueous metabolites). In order, results show greatest localization of metabolites from the central nervous system, brain, neuron, prostate, and mitochondria. Localizations with common metabolites are linked with edges.

## Supplementary Tables

| **Table S1.** Individual gastrointestinal symptom rating scale scores of IF1-P and IF2-P groups at baseline and week 4. | | | | |
| --- | --- | --- | --- | --- |
|  | **Baseline** | | **Week 4** | |
| **Symptom** | **IF1-P (*n* = 10)** | **IF2-P (*n* = 10)** | **IF1-P (*n* = 10)** | **IF2-P (*n* = 10)** |
| Stomach pain | 1.0 (1.0 – 5.0) | 1.0 (1.0 – 4.0) | 1.0 (1.0 – 3.0) | 1.0 (1.0 – 3.0) |
| Heartburn | 1.5 (1.0 – 5.0) | 1.5 (1.0 – 2.0) | 1.0 (1.0 – 2.0) | 1.0 (1.0 – 2.0) |
| Acid reflux | 1.5 (1.0 – 5.0) | 1.5 (1.0 – 3.0) | 1.0 (1.0 – 3.0) | 1.0 (1.0 – 2.0) |
| Hunger pains | 1.5 (1.0 – 4.0) | 2.0 (1.0 – 4.0) | 2.0 (1.0 – 3.0) | 2.0 (1.0 – 3.0) |
| Nausea | 1.0 (1.0 – 3.0) | 1.0 (1.0 – 3.0) | 1.0 (1.0 – 5.0) | 1.0 (1.0 – 2.0) |
| Rumbling | 2.0 (1.0 – 3.0) | 1.0 (1.0 – 2.0) | 3.0 (1.0 – 6.0) | 2.0 (1.0 – 5.0) |
| Bloating | 2.0 (1.0 – 4.0) | 2.0 (1.0 – 5.0) | 2.0 (1.0 – 4.0) | 1.0 (1.0 – 4.0) |
| Burping | 1.0 (1.0 – 2.0) | 1.0 (1.0 – 4.0) | 1.0 (1.0 – 2.0) | 1.0 (1.0 – 2.0) |
| Gas | 2.0 (1.0 – 5.0) | 1.5 (1.0 – 5.0) | 2.5 (1.0 – 6.0) | 1.0 (1.0 – 5.0) |
| Constipation | 1.0 (1.0 – 2.0) | 2.0 (1.0 – 6.0) | 2.0 (1.0 – 4.0) | 1.5 (1.0 – 4.0) |
| Diarrhea | 1.0 (1.0 – 5.0) | 1.0 (1.0 – 5.0) | 1.0 (1.0 – 1.0) | 1.0 (1.0 – 1.0) |
| Loose stools | 2.0 (1.0 – 4.0) | 1.0 (1.0 – 5.0) | 1.0 (1.0 – 1.0) | 1.0 (1.0 – 1.0) |
| Hard stools | 1.0 (1.0 – 2.0) | 2.0 (1.0 – 5.0) | 2.0 (1.0 – 3.0) | 1.0 (1.0 – 4.0)* |
| Urgent to defecate | 1.0 (1.0 – 5.0) | 1.0 (1.0 – 5.0) | 1.0 (1.0 – 3.0) | 1.0 (1.0 – 2.0) |
| Sensation of not completely emptying bowels | 1.5 (1.0 – 3.0) | 2.5 (1.0 – 5.0) | 3.0 (1.0 – 4.0) | 1.0 (1.0 – 3.0)* |
| *Significant difference in Δ score (post – pre score) vs IF1-P, Mann-Whitney U, *p* < 0.05. Data are presented as median and range. | | | | |

| **Table S2.** Fecal short-chain fatty acid (SCFA) concentrations of IF1-P and IF2-P groups at baseline and week 4. | | | | |
| --- | --- | --- | --- | --- |
|  | **Baseline** | | **Week 4** | |
| **SCFAs (mMol/g wet feces)** | **IF1-P (*n* = 10)** | **IF2-P (*n* = 10)** | **IF1-P (*n* = 10)** | **IF2-P (*n* = 10)** |
| Formate | 0.018 ± 0.017 | 0.085 ± 0.046 | 0.048 ± 0.043 | 0.038 ± 0.056 |
| Acetate | 0.126 ± 0.052 | 0.124 ± 0.094 | 0.118 ± 0.079 | 0.189 ± 0.139 |
| Propionate | 0.013 ± 0.006 | 0.014 ± 0.007 | 0.009 ± 0.004 | 0.015 ± 0.011 |
| Isobutyrate | 0.014 ± 0.008 | 0.019 ± 0.022 | 0.010 ± 0.003 | 0.014 ± 0.010 |
| Butyrate | 0.181 ± 0.098 | 0.192 ± 0.122 | 0.184 ± 0.069 | 0.210 ± 0.100 |
| Valerate | 0.016 ± 0.015 | 0.014 ± 0.017 | 0.020 ± 0.020 | 0.022 ± 0.017 |
| Values reported as mean ± SD. | | | | |

| **Table S3.** Adonis model parameters and term results for Bray-Curtis dissimilarity matrix. | | | | | |
| --- | --- | --- | --- | --- | --- |
|  | **DF** | **SS** | ***R*^2^** | ***F*** | ***p*** |
| **Group** | 1 | 0.435 | 0.041 | 3.416 | 0.001 |
| **Time** | 1 | 0.442 | 0.042 | 3.469 | 0.001 |
| **Subject** | 18 | 7.264 | 0.689 | 3.166 | 0.001 |
| **Group: Time** | 1 | 0.097 | 0.009 | 0.758 | 0.823 |
| **Residuals** | 18 | 2.294 | 0.218 |  |  |
| **Total** | 39 | 10.532 | 1.000 |  |  |
| Formula = bray.dm ~ group * time + subject. Number of permutations = 999.  Abbreviations: DF, degrees of freedom; SS, sum of squares. | | | | | |

| **Table S4.** Significance, fold change (FC), and area under the curve (AUC) details of metabolite markers of intermittent fasting. | | | |
| --- | --- | --- | --- |
| **Metabolite** | ***q**** | **FC**** | **AUC** |
| Serine | 0.003 | 1.25 | 0.71 |
| TMAO | 0.012 | 1.60 | 0.74 |
| Levulinic acid | 0.017 | 1.41 | 0.69 |
| 3-Aminobutyric acid | 0.029 | 1.32 | 0.65 |
| Citrate | 0.033 | 1.15 | 0.66 |
| Isocitrate | 0.033 | 1.15 | 0.66 |
| Glucuronic acid | 0.049 | 1.14 | 0.63 |
| *Derived from age-, body mass index-, sex-, and time-adjusted general linear model (GLM) with false discovery rate (FDR) correction.  **Analyzed as IF2-P/IF1-P. | | | |
